# 298. Host Response Profiling from Clinical Metagenomic Sequencing Data for Diagnosis of Central Nervous System Infections

**DOI:** 10.1093/ofid/ofae631.088

**Published:** 2025-01-29

**Authors:** Charles Omura, Mikael de Lorenzi-Tognon, Patrick Benoit, Venice Servellita, Kafaya Foresythe, Noah Brazer, Miriam Oseguera, Danielle Ingebrigtsen, Jessica Streithorst, Doug Stryke, Kelsey Zorn, Mary Karalius, Michael R Wilson, Charles Chiu

**Affiliations:** University of California San Francisco, San Francisco, California; University of California San Francisco, San Francisco, California; University of California San Francisco, San Francisco, California; University of California San Francisco, San Francisco, California; University of California San Francisco, San Francisco, California; University of California San Francisco, San Francisco, California; University of California San Francisco, San Francisco, California; University of California San Francisco, San Francisco, California; University of California San Francisco, San Francisco, California; University of California San Francisco, San Francisco, California; UCSF, San Francisco, CA; University of California San Francisco, San Francisco, California; UCSF, San Francisco, CA; UCSF, San Francisco, CA

## Abstract

**Background:**

Clinical metagenomic next-generation sequencing (mNGS) testing of cerebrospinal fluid (CSF) increases diagnostic yield for suspected central nervous system (CNS) infections. Nevertheless, up to 45% of cases remain unknown despite extensive testing and 6 months of follow-up. We developed artificial intelligence-machine learning (AI-ML) classification models (classifiers) based on RNA gene expression / host response data from CSF mNGS testing to enhance diagnostic performance.

**Figure 1.**
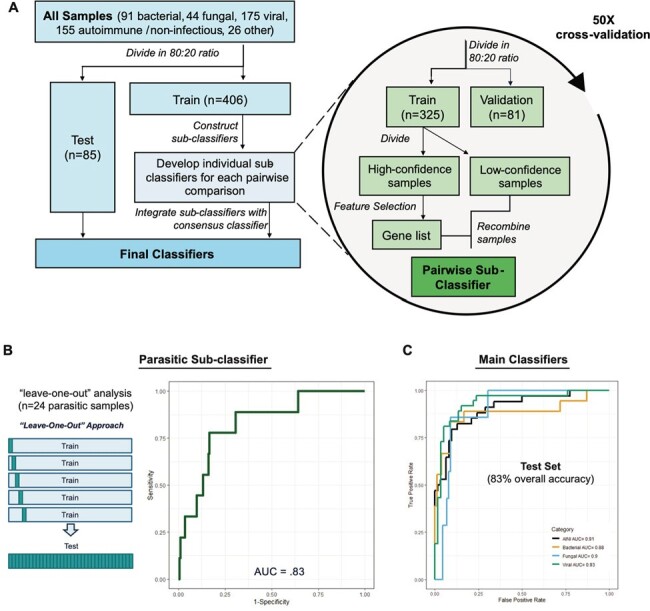
An artificial intelligence machine learning classifier. (A) Schematic illustration of the different steps to generate the sub-classifiers then integrated into a consensus classifier. (B) The parasitic sub-classifier was generated using the "leave-one-out" algorithm due to the low sample numbers. (C) Performance metrics of the main classifiers.

**Methods:**

From June 2016 and April 2023, 464 CSF mNGS results from UCSF patients with a confirmed viral (n=175), bacterial (n=91), fungal (n=44), or non-infectious (n=155) diagnosis were randomly divided into training and test subsets in an 80:20 ratio (Figure 1A). Clinicians adjudicated their confidence in the final diagnosis based on blinded medical chart review and laboratory test results. Gene (feature) selection was carried out using high-confidence samples, followed by 50-fold cross-validation. All possible pairwise comparisons were performed to generate sub-classifiers which were then integrated into a consensus classifier. Performance metrics were obtained by running the consensus classifier on the independent test subset. A separate classifier was developed for parasitic infections (n=24) using a “leave-one-out” algorithm to assess performance (Figure 1B). Classifier results were displayed as a score ranging from 0 to 10, corresponding to specificities of ≤70% to ≥99%.

**Figure 2.**
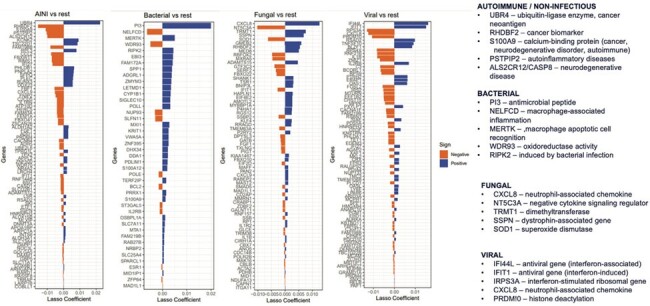
Differentially expressed gene analysis with pair-wise comparison between sub-classifiers. Left panel shows genes selected by the LASSO (least absolute shrinkage and selection operator) algorithm, and right panel the pathway/function these genes.

**Results:**

Classifier accuracy based on the test set was 83%, with individual area under the curve (AUC) scores ranging from 0.88-0.93 for each category (Figure 1C). The function of the selected genes corresponded well with the category (Figure 2). Classifier examples include patients with (i) culture-negative CNS tuberculosis, (ii) persistent

*Toxoplasma gondii*

infection despite 19 days of treatment, (iii) a rare autoimmune syndrome, and (iv) a chronic enterovirus infection misclassified as an atypical bacterial infection, suggesting that acute and chronic host response profiles differ (Figure 3).

**Figure 3.**
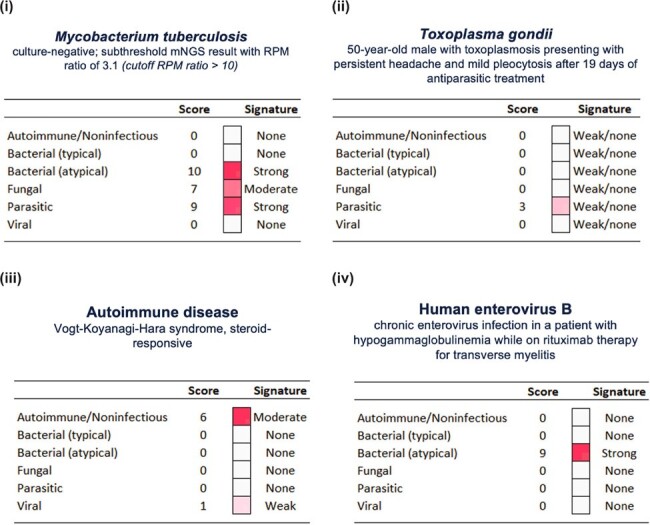
Classifier examples. (i) culture-negative CNS tuberculosis, (ii) persistent Toxoplasma gondii infection despite 19 days of treatment, (iii) a rare autoimmune syndrome, and (iv) a chronic enterovirus infection misclassified as an atypical bacterial infection.

**Conclusion:**

Host response profiling with AI-ML classifiers complements mNGS testing and can enhance diagnostic yield for unexplained CNS syndromes.

**Disclosures:**

**Charles Chiu, MD, PhD**, Abbott Laboratories, Inc: Grant/Research Support|Biomeme: Advisor/Consultant|Biomeme: Board Member|BiomeSense: Advisor/Consultant|BiomeSense: Board Member|Delve Bio: Advisor/Consultant|Delve Bio: Board Member|Delve Bio: Grant/Research Support|Flightpath Biosciences: Advisor/Consultant|Flightpath Biosciences: Board Member|Mammoth Biosciences: Advisor/Consultant|Mammoth Biosciences: Board Member|Pathogen detection using next generation sequencing: US patent 11380421|Poppy Health: Advisor/Consultant|Poppy Health: Board Member

